# Rare Case of Primary Pulmonary Pleomorphic Liposarcoma Treated With Multimodal Therapy

**DOI:** 10.31486/toj.20.0164

**Published:** 2021

**Authors:** Treshita Dey, Divya Khosla, Divyesh Kumar, Debajyoti Chatterjee, Renu Madan, Harjeet Singh, Harkant Singh, Rakesh Kapoor

**Affiliations:** ^1^ Department of Radiotherapy and Oncology, Postgraduate Institute of Medical Education and Research, Regional Cancer Centre, Chandigarh, India; ^2^ Department of Histopathology, Postgraduate Institute of Medical Education and Research, Chandigarh, India; ^3^ Department of Surgical Gastroenterology, Postgraduate Institute of Medical Education and Research, Chandigarh, India; ^4^ Department of Cardiovascular and Thoracic Surgery, Postgraduate Institute of Medical Education and Research, Chandigarh, India

**Keywords:** *Liposarcoma*, *peritoneal metastasis*, *primary pulmonary sarcoma*

## Abstract

**Background:** Pleomorphic liposarcoma (PLS) is a very rare type of primary pulmonary sarcoma. Clinical data about these tumors are limited, and optimal treatment has not yet been defined.

**Case Report:** A 32-year-old male presented with pain and heaviness over the right chest. Contrast-enhanced computed tomography (CECT) of the chest showed a heterogeneous hypodense pleural-based mass and a mediastinal mass. The patient was treated with chemoradiotherapy, followed by excision and adjuvant chemotherapy. Seven months after completion of treatment, he presented with an abdominopelvic mass and soft tissue peritoneal deposits. The mass was resected, and second line chemotherapy resulted in a partial response. The patient was routinely followed. Six months after completion of the second round of chemotherapy, CECT showed multiple soft tissue deposits in the right lumbar region, right hemipelvis, and presacral region with no evidence of pulmonary disease. Chemotherapy elicited a partial response. Three years from the date of diagnosis, the patient was alive with stable disease.

**Conclusion:** This case is unique because of the rare primary site of PLS presentation and the rare presentation of peritoneal metastasis. Citing such cases would help us to define adequate treatment protocols for this aggressive tumor.

## INTRODUCTION

Liposarcoma is a relatively common soft tissue sarcoma, but primary pulmonary liposarcoma is a rare neoplasm. Primary pulmonary sarcomas are extremely rare and account for <1% of all lung tumors.^1^ Pleomorphic liposarcoma (PLS) is a pleomorphic, highly malignant liposarcoma, with a unique arrangement of sheets of lipoblasts in histopathology. PLS is rare, accounting for only 5% to 10% of all lipomatous tumors.^2^ PLS is characterized by a high rate of local invasion, metastasis, and recurrence with an aggressive clinical course and is therefore considered to be the most malignant of the liposarcomas.^3^ To the best of our knowledge, only 15 cases of primary pulmonary liposarcoma had been reported as of December 2020.^3-14^ Of these 15 cases, only 4 patients had PLS histology.^3,9,11,14^

We present a case of primary pulmonary PLS with a rare pattern of peritoneal metastasis that was managed with multimodal treatment.

## CASE REPORT

**Figure 1.**
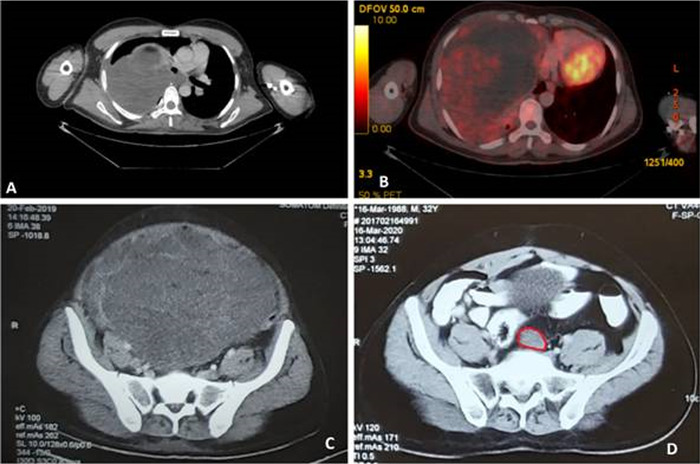
(A) Contrast-enhanced computed tomography (CECT) shows pleural-based heterogenous hypodense mass. (B) Baseline positron emission tomography–computed tomography shows local disease. (C) CECT shows large abdominopelvic mass. (D) CECT shows presacral deposit.

A 32-year-old male presented in March 2017 with complaints of pain and heaviness over the right chest for 2 months, aggravated by coughing and deep inspiration. The patient also reported shortness of breath on exertion. He was a chronic smoker (5 pack-years) and binged on alcohol on occasion. Clinically, breath sounds were absent over the right basal zone. Chest x-ray revealed homogenous opacity involving the right hemithorax. Baseline contrast-enhanced computed tomography (CECT) of the chest revealed a well-defined 20.8 × 13.6 × 13-cm pleural-based heterogeneous hypodense mass (10-12 Hounsfield units) along the right lateral chest wall, medially extending into the mediastinum and compressing the heart with collapse of the right lung (Figure 1A). Bronchoscopy showed distorted anatomy of the right tracheobronchial tree with extrinsic airway compression. Computed tomography (CT)-guided core needle biopsy from the pleural-based mass identified PLS. No mass was palpable in the abdomen and extremities. Baseline positron emission tomography-CT (PET-CT) for staging revealed localized disease (Figure 1B), so the likelihood of metastasis from the common primary sites of liposarcoma (eg, extremities, retroperitoneum) was excluded.

The diagnosis of primary PLS of the lung was made after PET-CT and the extensive immunohistochemistry panel on the biopsy specimen ruled out the possibility of other primary sites. The tumor was deemed inoperable because of its large size and the disease extension, so the patient was considered for chemoradiotherapy. To downsize the tumor, the patient was started with a regimen of vincristine (1.4 mg/m^2^, maximum dose capped at 2 mg)/doxorubicin (75 mg/m^2^)/cyclophosphamide (1,200 mg/m^2^ with mesna) every 3 weeks. Response assessment after 3 cycles via CECT revealed no substantial change in tumor size, and the patient then underwent hypofractionated radiotherapy to the lung mass with a dose of 30 Gy in 10 fractions for 2 weeks, with 6 MV photons using parallel-opposed fields.

A partial response was noted after radiotherapy, and the patient underwent excision of the tumor via the median sternotomy and anterolateral thoracotomy approach. Postoperative histopathology showed PLS with focal myxoid changes and large areas of necrosis. A specimen of the diaphragm showed tumor infiltration. After surgery, the patient received 6 cycles of ifosfamide (1,800 mg/m^2^ with injections of mesna 400 mg/m^2^ at 0, 4, and 8 hours)/etoposide (100 mg/m^2^) every 3 weeks. The patient tolerated the therapy well, and posttreatment CECT at 6 weeks revealed no residual disease. The patient was followed up with clinical examinations every 2 months, and CECT at 3 months did not show any evidence of disease.

Seven months after completion of chemotherapy, the patient presented with complaints of fullness and tenderness in the right iliac fossa, persistent constipation, and vomiting not relieved by conservative management. CECT of the abdomen revealed a 30.5 × 25-cm abdominopelvic mass and 2 soft-tissue deposits in the subhepatic space and rectovesical pouch with no residual disease in the chest (Figure 1C).

**Figure 2.**
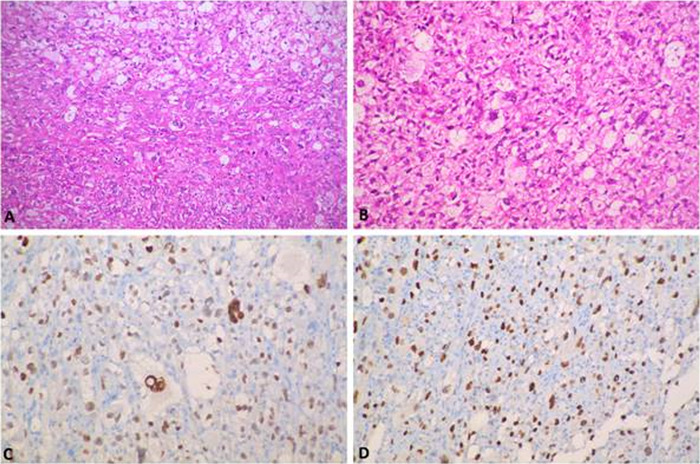
(A) Pleomorphic liposarcoma tumor deposit shows admixture of pleomorphic lipoblasts with epithelioid cells (hematoxylin and eosin, ×100). (B) Pleomorphic liposarcoma tumor deposit shows multivacuolated lipoblasts with indented pleomorphic nuclei (hematoxylin and eosin, ×200). (C) Pleomorphic liposarcoma cells are positive for S-100 (immunohistochemistry, ×200). (D) Pleomorphic liposarcoma cells show diffuse p53 expression (immunohistochemistry, ×200).

The patient underwent resection of an abdominopelvic mass that occupied the entire abdomen. The tumor was encapsulated, solid (lipomatous), and cystic (hemorrhagic) with capsule rupture, and it was removed in pieces. The histopathology of both the thoracic and abdominal tumors showed features of PLS (Figures 2A and 2B) with brisk mitosis (18/10 high power fields) and necrosis (<50%). The tumor was composed of numerous pleomorphic multivacuolated lipoblasts admixed with epithelioid malignant cells. All resection margins were free, and the specimen was labeled as Fédération Nationale des Centres de Lutte Contre le Cancer (FNCLCC) grade III (tumor differentiation score 3, mitosis score 2, tumor necrosis score 1). Immunohistochemistry showed diffuse nucleocytoplasmic positivity for S100 calcium-binding protein P and nuclear expression of p53 in 80% of cells but negativity for MDM2 (Figures 2C and 2D). Postoperative CECT showed peritoneal and omental deposits.

The patient was treated with 3 cycles of gemcitabine (1,000 mg/m^2^ on days 1 and 8)/docetaxel (75 mg/m^2^) every 3 weeks. Interval response evaluation according to the Response Evaluation Criteria in Solid Tumors (RECIST) showed partial response, and chemotherapy was continued for 3 more cycles. Imaging after treatment completion showed stable disease in the abdomen with peritoneal deposit. Because the patient was asymptomatic, no further active treatment was planned, and the patient was followed with clinical examinations every 2 months and radiologic evaluations every 3 months.

Six months after completion of the second round of chemotherapy, the patient presented with intermittent pain over the right iliac fossa. CECT showed multiple soft tissue deposits in the right lumbar region, right hemipelvis, and presacral region with no evidence of pulmonary disease (Figure 1D). The patient completed 6 cycles of single-agent chemotherapy with liposomal doxorubicin (50 mg/m^2^) every 4 weeks. Two months after completion of chemotherapy, CECT showed partial response.

Three years from the date of diagnosis, the patient was alive with stable disease. During the treatment period, he received 4 cycles of chemotherapy.

## DISCUSSION

Sarcomas constitute <1% of all pulmonary malignancies, and PLS is the least common variant.^1,4^ The earliest reported case of primary pulmonary liposarcoma was in the 1940s by Latienda and Itoiz.^5^ Liposarcomas originate from primitive mesenchymal cells and are common in the extremities or retroperitoneum. For primary intrathoracic liposarcoma, the mediastinum is the most common site of origin; primary liposarcomas rarely arise from lung parenchyma, chest wall, or pleura.^3^ Hochberg and Crastnopol analyzed 77 patients with primary pulmonary sarcoma, and the most common variants observed were fibrosarcoma and leiomyosarcoma, with liposarcoma seen in only 2 patients.^6^ The Table presents a review of the literature of primary pulmonary PLS, as well as the details of this case.

**Table.** Pleomorphic Liposarcoma Cases Reported in the Literature With Treatment and Outcome Details

Study	Age, years	Surgery	Radiotherapy	Chemotherapy	Pattern of Recurrence	Outcome
Krygier et al, 1997^9^	49	Lobectomy	Yes (40 Gy)	Yes	Bone metastasis	Died at 8 months
Ibe et al, 2005^11^	36	Lobectomy	No	No	Local	Died at 2 months
Achir et al, 2009^14^	49	Pneumonectomy	No	No	No recurrence	Alive at 3 years
Chen et al, 2014^3^	49	Complete resection	Yes	Yes	Bone metastasis	Alive at ≥16 months
Our case	32	Complete resection	Yes (30 Gy)	Yes	Peritoneal metastasis	Alive at 3 years

Based on published cases, primary pulmonary liposarcomas occur in patients with a wide age range (9 to 61 years) and a median age of 46.5 years, present earlier than lung cancer, and have no sex predilection. Our patient presented at 32 years of age. Possible risk factors associated with development of primary pulmonary liposarcoma are pleuropulmonary asbestosis or malignant transformation of pulmonary lipoma.^13^ However, in our case, no such risk factors were identified. Primary pulmonary liposarcomas present with symptoms similar to those of lung cancer, such as cough, sputum production, hemoptysis, dyspnea, chest pain, and loss of weight and appetite. Because of similar symptomatology, primary pulmonary liposarcoma may be misdiagnosed as pulmonary tuberculosis in developing countries such as India because of the high prevalence of that disease.^8^ Approximately 10% of all primary pulmonary liposarcomas are asymptomatic, which may be because of the expansile nature of the tumor rather than infiltration.^6^ Also, because these tumors are slow growing, they often present as a large mass. The absence of any characteristic symptom further delays diagnosis.

Primary pulmonary liposarcoma can primarily involve the lung but can also be completely pleural in origin, such that in advanced cases, distinguishing a pulmonary from a pleural mass can be difficult. Usually, these tumors involve lung parenchyma and therefore are not visualized during bronchoscopy. Only 2 cases of primary pulmonary liposarcoma have been reported with positive findings on bronchoscopy, seen as polypoid masses and causing near total obstruction.^4,9^ In this case, we observed extrinsic compression of the tracheobronchial tree, but no exophytic mass could be appreciated on bronchoscopy.

Said et al described the radiologic findings of primary pulmonary liposarcoma: a well-defined lobulated inhomogeneous hypodense mass interspersed with multiple areas of very low densities, suggestive of fat.^10^ Also, on magnetic resonance imaging, the mass showed high signal intensity on T2-weighted images and low intensity with fat-saturated T2-weighted images. In our case, CT showed similar findings. Therefore, the differential diagnosis of primary pulmonary liposarcoma is possible from imaging. Peritoneal dissemination has been cited only once in a case of undifferentiated pleomorphic sarcoma.^15^ To the best of our knowledge, the extensive dissemination seen in our case has not been reported before in a case of primary pulmonary PLS.

Liposarcomas are categorized into 4 broad histologic groups. Well-differentiated liposarcomas (also known as atypical lipomatous tumors) have a better prognosis compared to myxoid/round cell–variant (intermediate prognosis), dedifferentiated, and pleomorphic tumors, which have poor prognoses as per the 2012 National Comprehensive Cancer Network classification.^3^ On immunohistochemistry, well-differentiated, myxoid/round-cell, and dedifferentiated liposarcomas are positive for vimentin, S-100, MDM2, and CDK4. PLS is usually negative for MDM2 and CDK4 but expresses p53. PLS resembles a malignant fibrous histiocytosis-like tumor, characterized by the predominance of pleomorphic spindle-shaped cells with occasional giant multinucleated cells.^16^ Fletcher emphasized the difficulty in distinguishing PLS from other high-grade pleomorphic sarcomas in his review of 159 such tumors; 21% were reclassified as PLS on review.^17^

Chen et al studied various prognostic factors for primary intrathoracic liposarcoma.^3^ They observed that tumor size, distinct tumor locations, surgery, age at presentation, and sex had no impact on overall survival (OS) and disease-free survival (DFS). Radical surgery had a significant effect on OS, and histologic subtype also had significant impact on both OS and DFS.^3^ However, when compared with other primary sites, pulmonary primaries had a worse prognosis. Despite negative surgical margins, early disease dissemination has been observed, probably because of microrupture or frank rupture observed during dissection of cystic masses. Disseminated disease was seen in our patient as well, with capsule rupture and the tumor having to be removed in pieces, which may have led to peritoneal seeding.

Treatment options for primary pulmonary liposarcoma are based on the algorithms used for management of primary liposarcomas in the extremities and retroperitoneum. Radical excision with lymph node dissection is the preferred treatment modality, and best survival is reported with R0 resection.^14^ Chen et al analyzed 23 cases of primary intrathoracic liposarcoma and observed that patients having radical surgery had significantly better OS (*P*=0.029) than those without surgery.^3^ They also reported that metastasis was frequently seen, predominantly in the lung, pleura, liver, and bone, most commonly of the dedifferentiated tumor type.

Chemotherapy and radiotherapy as adjuvant treatment in those with residual disease, inoperable cases, or metastasis have shown limited success in soft-tissue sarcomas. Doxorubicin when given alone or in combination with ifosfamide results in significant response in sarcomas.^18^

In 2016, Nassif et al discussed advances in the management of liposarcomas—predominantly primaries of the extremities and retroperitoneum—but the same management can be tried in pulmonary primaries.^19^ In sarcomas of the extremities, adjuvant radiotherapy after limb-sparing surgery improved local control. This improved control with adjuvant radiotherapy can be extrapolated to pulmonary liposarcomas. Myxoid liposarcomas are radiosensitive, and excellent responses have been observed with neoadjuvant radiotherapy.^20^ However, radiotherapy improves only local control with no improvement in survival.^19^

Liposarcomas of the extremities show good response to systemic chemotherapy. For metastatic disease, the combination of ifosfamide/doxorubicin or gemcitabine/docetaxel results in a 16% to 43% response and OS of 18 to 30 months.^21,22^ The US Food and Drug Administration has approved trabectedin (especially for myxoid/round-cell histology) and eribulin (principally for the dedifferentiated and pleomorphic variants) as second-line agents for treatment of liposarcoma.^23-25^

## CONCLUSION

Primary pulmonary PLS is an extremely rare neoplasm. This case is unique because of the rare primary site of PLS presentation and the rare presentation of peritoneal metastasis. PLS behaves as a high-grade tumor with frequent metastasis. Because limited data are available, standard treatment guidelines have not been formulated, and treatment is principally extrapolated from experience in treating liposarcomas of the extremities or retroperitoneum. Reporting more cases would help us understand the natural behavior of these aggressive malignancies to optimize treatment options and follow-up.

## Notes

*This article meets the Accreditation Council for Graduate Medical Education and the American Board of Medical Specialties Maintenance of Certification competencies for Patient Care and Medical Knowledge.*
